# Ossification of the yellow ligament in the cervical spine – an unusual location

**DOI:** 10.1051/bmdcn/2019090214

**Published:** 2019-05-24

**Authors:** Jacob Yoong-Leong Oh, Victor Tzong-Jing Wang, Timothy Wei Wen Teo, Arun-Kumar Kaliya-Perumal, Hwan Tak Hee

**Affiliations:** 1 Department of Orthopaedic Surgery, Spine Division, Tan Tock Seng Hospital Singapore; 2 Department of Orthopaedic Surgery, Khoo Teck Puat Hospital Singapore; 3 Department of Orthopaedic Surgery, Melmaruvathur Adhiparasakthi Institute of Medical sciences and Research, Affiliated to the Tamil Nadu Dr MGR Medical University Tamil Nadu India; 4 Pinnacle Spine and Scoliosis Centre, Mount Elizabeth Medical Centre Singapore

**Keywords:** Ligamentum flavum, Neurologic manifestations, Ossification, Spinal cord compression, Spine

## Abstract

Ossification of the yellow ligament (OYL) or ligamentum flavum, usually occurs in the thoracic spine. Focal OYL occurring in the cervical spine is considered rare and is sparsely reported in the literature. We came across a 30-year-old male patient with progressive left upper limb and bilateral lower limb weakness over a period of 3 months, associated with an unsteady gait. Clinical examination revealed bilateral generalized hyper-reflexia in both upper and lower limbs, inverted supinator jerk, Hoffman’s sign and clonus. Myelopathy due to cord compression was suspected and further investigations were done. MRI and CT scans revealed a bony mass in relation to the C6 spinous process projecting anterosuperiorly and narrowing the cervical spinal canal causing cord signal changes from C4 to C6 levels. In view of the deteriorating neurological status, immediate surgery in the form of decompression and posterior stabilization from C4-C6 was performed. Patient gradually recovered after surgery and attained full functional status. We report this case considering the unusual location of OYL and its successful management.

## 1. Introduction

Ossification of the yellow ligament (OYL), which is also known as the ligamentum flavum, is an uncommon cause of cervical cord compression. The condition was first reported by Polgar in 1920 [1]; since then, many similar reports dealing with thoracic OYL have been published [2–7]. OYL in the cervical spine is relatively rare with only a few cases reported. Most of these reports depict the occurrence of OYL in elderly patients of East Asian ancestry, particularly the Japanese.

The reason for predisposition of the thoracic spine and frequent association with thoracic prolapsed intervertebral disc herniation in the same spinal segment remains unclear and believed to be of mechanical origin. The condition is slowly progressive and usually requires surgical intervention to prevent subsequent neurological compromise. This article presents a case of cervical myelopathy secondary to OYL in a young African patient. The clinical features, pathogenesis, diagnosis and management are discussed in this report.

## 2. Case report

A 30-year old male engineer of African descent, presented with progressive left upper limb and bilateral lower limb weakness over a period of 3 months, associated with an unsteady gait. He complained of altered sensation of his feet, which he described as ‘walking on cotton wool’. He denied any preceding trauma and there was no neck or back pain.

Clinical examination revealed that he had normal power in both upper and lower limbs. However, there was generalized hyper-reflexia in all muscle groups of both upper and lower limbs, bilateral inverted supinator jerk, positive Hoffman’s sign and clonus. Sensation was decreased in the left sole of the foot sparing the dorsum. At the time of presentation, patient had a Japanese Orthopaedic Association (JOA) score of 7 and significant gait disability (Grade 3 as per Nurick’s Grading) [8].

**Fig. 1:**
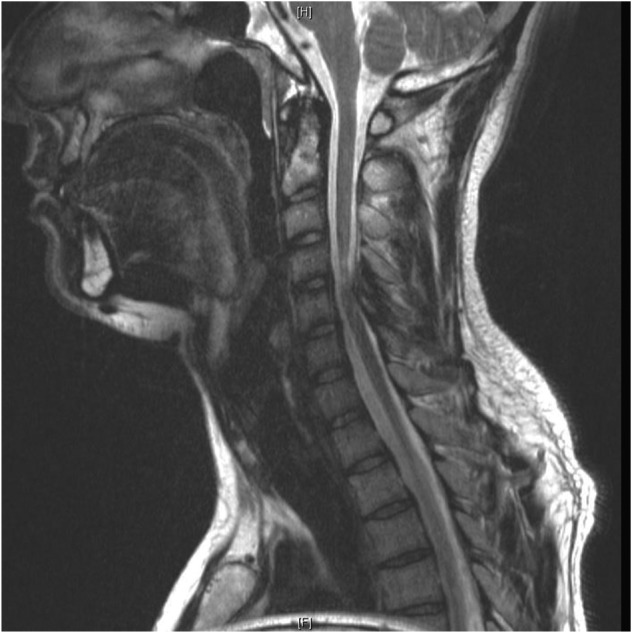
Sagittal T2 weighted MR image of the cervical spine showing a narrowed spinal canal due to a low signal intensity mass anterior to the posterior elements with cord oedema at C4- C6 levels.

**Fig. 2:**
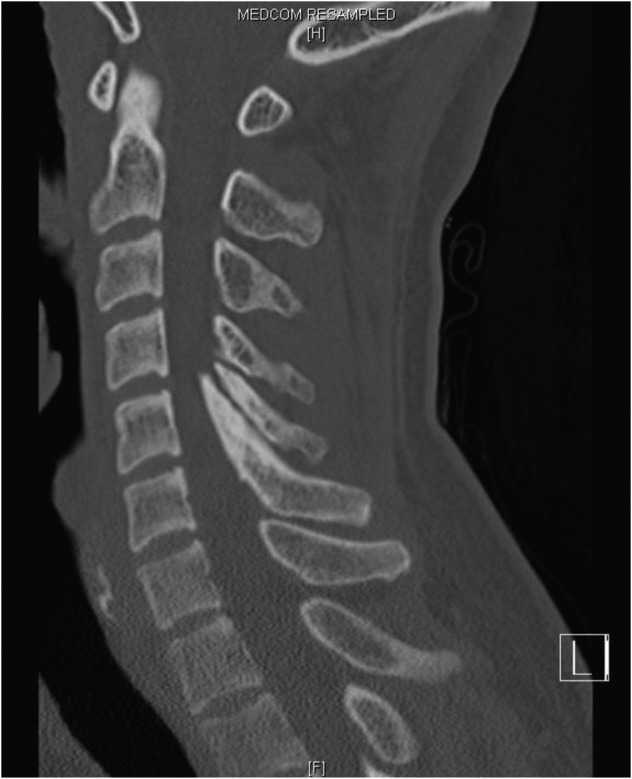
Sagittal computed tomography image of the cervical spine showing a beak like outgrowth from the posterior elements protruding into the spinal canal, the classical appearance of an ossified yellow ligament.

**Fig. 3:**
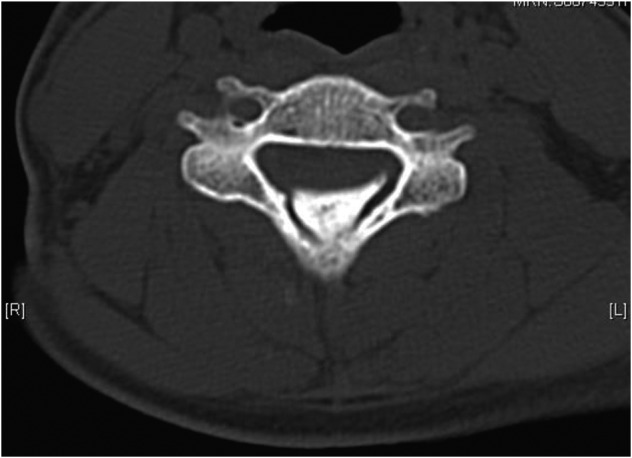
Axial computed tomography image showing thickened nodular ossified yellow ligament centred at C5 level.

Magnetic Resonance Imaging (MRI) of the whole spine showed stenosis with cord signal changes from C4 to C6 levels, secondary to impingement by a prominent bony growth arising antero-superiorly from the C6 spinous process and indenting the spinal canal (Fig. 1). Computed Tomography of the cervical spine further confirmed the bony mass in relation to the C6 spinous (Fig. 2, 3).

**Fig. 4:**
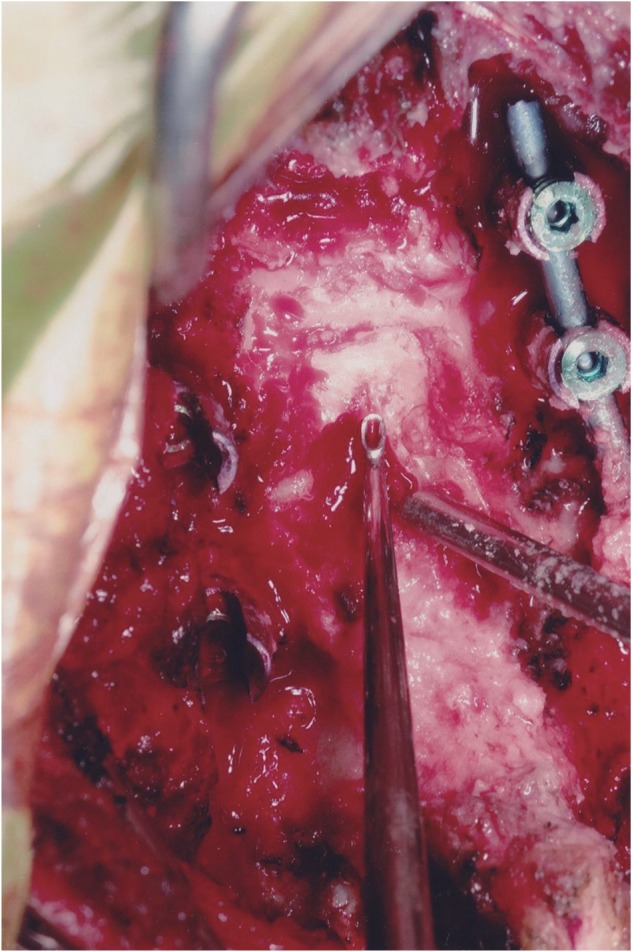
Intraoperative photograph of the surgical field, after posterior instrumentation and laminectomy, showing the ossified segment of the yellow ligament pointed with the curette.

**Fig. 5:**
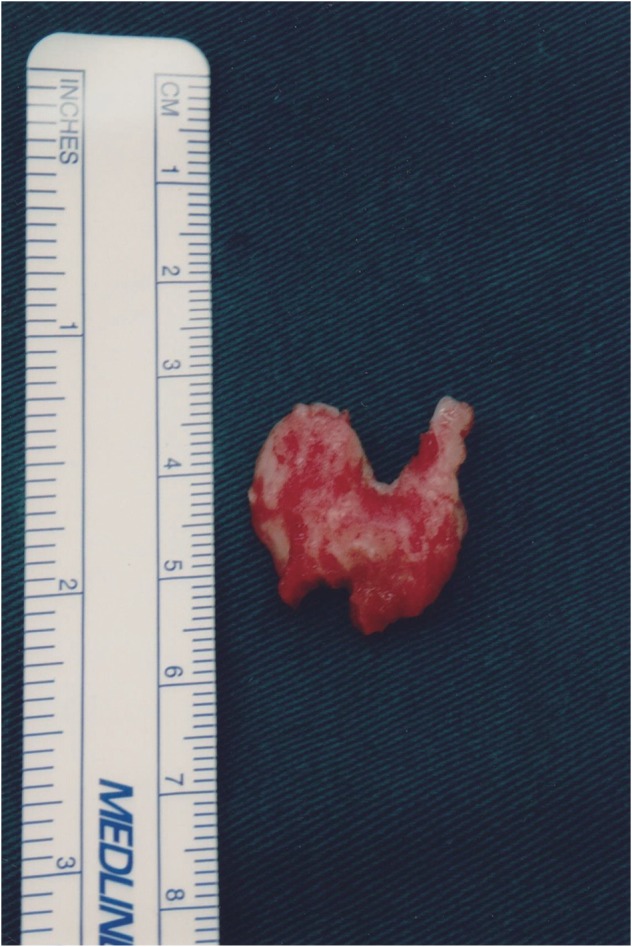
A bigger chunk of the ossified yellow ligament extracted during surgery.

In view of the above findings, cervical myelopathy resulting from spinal canal stenosis due to OYL was suspected. A posterior decompression laminectomy with excision of the ossified yellow ligament along with posterior instrumentation and fusion from C4-C6 was planned. Lateral mass screws were inserted into C4, C5 and C6. A wide Laminectomy was performed and the OYL was identified. With help of a burr, OYL was freed and removed in a piecemeal manner. OYL was found to be adherent to the dura and care was taken to ensure there was no dural tear and CSF leak (Fig. 4, 5).

Histological examination of the surgical specimen showed endochondral ossification, lamellar bone and marrow formation within the bony tissue. This was consistent with our diagnosis of OYL. Postoperative period was uneventful, and rehabilitation was initiated as tolerated. Subjective weakness and unsteadiness resolved eventually, and patient recovered to his full functional status by 6 months.

## 3. Discussion

Compressive myelopathy secondary to ossification of the yellow ligament is an uncommon phenomenon with only a few documented cases in the literature. More than 95% of these cases were reported from Asia, in particular, among the East Asian population. The most common site of occurrence is the thoracic spine leading to thoracic myelopathy [3–6, 9]. A recent CT based large cross- sectional study among the Japanese population observed that the distribution of OYLs in the thoracic spine was common at T10-T11 and T4-T5 levels [10]. Another MRI-based crosssectional study on 1736 patients from China found cervical spine involvement in only 4% of the patients, mostly in association with lesions in other parts of the spine [11]. Our patient is unique due to the presentation of a solitary OYL at the cervical spine.

From a surgical perspective, it is important to differentiate ossification from calcification. In OYL, there is formation of mature bone in the ligamentum flavum, characterized by the growth and replacement of the flavum with lamellar bone. In calcification of the yellow ligament (CYL), there is an amorphous calcium crystal deposition within the ligamentum flavum, leading to calcification but no formation of mature bone [12, 13].

In OYL, ossification begins from the capsular part of the flavum, usually forming 2 nodular masses before progressing towards the interlaminar portion at the midline. As the ossification continues, there is unification of adjacent laminae; following which, canal compromise begins from the posterior aspect [12, 14]. This is important as reports have revealed that there is a greater chance that the ossified ligament will be adherent to the dura, resulting in a higher risk of dural tears. However, this is not so apparent in patients with CYL [5-7, 17-18].

During the surgery, we performed a wide laminectomy combined with stabilization of the cervical spine with lateral mass screws. A wider surgical field allows for better decompression and visualization of normal anatomy before “peeling off’ the adherent OYL from the underlying dura. Instrumentation and fusion allows for stabilization of the posteriorly decompressed levels and also eradicates the hypermobility which might have been an aetiological factor in the development of the condition [4–6, 9].

## 4. Conclusion

This case report highlights an uncommon occurrence of a solitary OYL in the cervical spine of a young patient who presented with cervical myelopathy. The combination of CT and MR imaging improves diagnostic accuracy. Wide decompression laminectomy along with stabilization is needed to safely address this pathology. Meticulous care is advised during peeling off the OYL from the adherent dura to prevent possible dural tear and CSF leak.

## References

[1] Polgar F. Uberinter akuellewirbelverkal kung. Fortschr Geb Rongenstr Nuklearmed Erganzungsband. 1920; 40: 292–8.

[2] Yamaguchi H, Tamagake S, Fujita S. A case of ossification of the ligamentum flavum with spinal cord tumor symptoms. Seikei Geika. 1960; 11: 951–6.

[3] Yonenobu K, Ebara S, Fujiwara K, Yamashita K, Ono K, Yamamoto T, *et al.* Thoracic myelopathy secondary to ossification of the spinal ligament. J Neurosurg. 1987; 66: 511–8. 3104552 10.3171/jns.1987.66.4.0511

[4] Kruse JJ, Awasthi D, Harris M, Waguespack A. Ossification of the ligamentum flavum as a cause of myelopathy in North America: report of three cases. J Spinal Disord. 2000; 13: 22–5. 10710144 10.1097/00002517-200002000-00004

[5] Kuh SU, Kim YS, Cho YE, Jin BH, Kim KS, Yoon YS. Contributing factors affecting the prognosis surgical outcome for thoracic OLF. Eur Spine J. 2006; 15: 485–91. 15902507 10.1007/s00586-005-0903-9 PMC3489313

[6] Li F, Chen Q, Xu K. Surgical treatment of 40 patients with thoracic ossification of the ligamentum flavum. J Neurosurg Spine. 2006; 4: 191–7. 16572617 10.3171/spi.2006.4.3.191

[7] Maiuri F, Iaconetta G, Gambardella A. Ossification of the yellow ligament causing thoracic cord compression. Arch Orthop Trauma Surg. 2000; 120: 346–8. 10853911 10.1007/s004020050480

[8] Nurick S. The pathogenesis of the spinal cord disorder associated with cervical spondylosis. Brain. 1972; 95: 87–100. 5023093 10.1093/brain/95.1.87

[9] Okada K, Oka S, Tohge K, Ono K, Yonenobu K, Hosoya T. Thoracic myelopathy caused by ossification of the ligamentum flavum. Clinicopathologic study and surgical treatment. Spine (Phila Pa 1976). 1991; 16(3): 280–7. 1902988 10.1097/00007632-199103000-00005

[10] Mori K, Kasahara T, Mimura T, Nishizawa K, Murakami Y, Matsusue Y, *et al.* Prevalence, distribution, and morphology of thoracic ossification of the yellow ligament in Japanese: results of CTbased cross-sectional study. Spine (Phila Pa 1976). 2013; 38(19): E1216–22. 24509558 10.1097/BRS.0b013e31829e018b

[11] Guo JJ, Luk KD, Karppinen J, Yang H, Cheung KM. Prevalence, distribution, and morphology of ossification of the ligamentum flavum: a population study of one thousand seven hundred thirty-six magnetic resonance imaging scans. Spine (Phila Pa 1976). 2010; 35(1): 51–6. 20042956 10.1097/BRS.0b013e3181b3f779

[12] Miyasaka K, Kaneda K, Sato S, Iwasaki Y, Abe S, Takei H, *et al.* Myelopathy due to ossification or calcification of the ligamentum flavum: radiologic and histologic evaluations. AJNR Am J Neuroradiol. 1983; 4(3): 629–32. 6410817 PMC8334879

[13] McCarty OJ Jr, Kohn NN, Faires JS. The significance of calcium phosphate crystals in the synovial fluid of arthritic patients: the “ pseudogout syndrome” I. Clinical aspects. Ann Intern Med. 1962; 56: 711–32. 10.7326/0003-4819-56-5-738 14457846

[14] Sato T, Tanaka Y, Aizawa T, Koizumi Y, Kokubun S. Surgical treatment for ossification of ligamentum flavum in the thoracic spine and its complications. Spine Spinal Cord. 1998; 11: 505–10.

[15] Couto AR, Brown MA. Genetic factors in the pathogenesis of CPPD crystal deposition disease. Curr Rheumatol Rep. 2007; 9(3): 231–6. 17531177 10.1007/s11926-007-0037-7

[16] Pascal-Moussellard H, Cabre P, Smadja D, Kaidomar S, Catonne Y. Myelopathy due to calcification of the cervical ligamenta flava: a report of two cases in West Indian patients. Eur Spine J. 1999; 8(3): 238–40. 10413352 10.1007/s005860050165 PMC3611172

[17] Kawano N, Matsuno T, Miyazawa S, Iida H, Yada K, Kobayashi N, *et al.* Calcium pyrophosphate dihydrate crystal deposition disease in the cervical ligamentum flavum. J Neurosurg. 1988; 68(4): 613–20. 2832553 10.3171/jns.1988.68.4.0613

[18] Oka S. Scanning electron microscopic observation of ossification and calcification of the Ligamentum flavum. Arch Jpn Chir. 1982; 51: 671–94. 6818908

